# Arabidopsis Serine Decarboxylase 1 (SDC1) in Phospholipid and Amino Acid Metabolism

**DOI:** 10.3389/fpls.2018.00972

**Published:** 2018-07-31

**Authors:** Yu-chi Liu, Farrel Gunawan, Ian Sofian Yunus, Yuki Nakamura

**Affiliations:** Institute of Plant and Microbial Biology, Academia Sinica, Taipei, Taiwan

**Keywords:** phospholipid biosynthesis, phospholipid, serine decarboxylase, glycerolipid metabolism, *Arabidopsis thaliana*

## Abstract

*Arabidopsis thaliana* serine decarboxylase 1 (SDC1) catalyzes conversion of serine to ethanolamine, the first reaction step of phosphatidylcholine and phosphatidylethanolamine biosynthesis. However, an involvement of SDC1 in amino acid metabolism remains elusive despite that serine is the substrate of SDC1. Here, we showed that SDC1 localizes in mitochondria although phosphatidylcholine and phosphatidylethanolamine are known to be produced in the endoplasmic reticulum (ER). Moreover, we found that overexpression of SDC1 decreased levels of amino acid compounds derived from mitochondrial tricarboxylic acid cycle. These results suggest that mitochondria-localized SDC1 plays an important role in both phospholipid and amino acid metabolism in *A. thaliana*.

## Introduction

Phospholipid is an essential component of biological membranes in most organisms (Vance, 2015). Its biosynthesis consists of an assembly of diacylglycerol backbone and polar head group (Nakamura, 2017). In plants, whereas diacylglycerol backbone is known to be synthesized from the glycolysis pathway, the initial reaction steps of the biosynthesis of polar head group are poorly studied (Lin et al., 2015; Nakamura, 2017). Animal phospholipid biosynthesis partially relies on exogenous uptake of precursory compounds through the food intake. For example, choline is an essential nutrient and its uptake readily provides a substrate for the biosynthesis of phosphatidylcholine (PC), the most abundant phospholipid class (Li and Vance, 2008). In autotrophic plants, supply of the precursors for the biosynthesis of polar head group must absolutely rely on their own biosynthesis. This difference highlights an importance of investigating polar head group biosynthesis in plant phospholipid metabolism.

Ethanolamine is an initial precursor for the biosynthesis of two primary phospholipid classes, PC and phosphatidylethanolamine (PE) (Lin et al., 2015). Plants have an unique enzyme activity that produces ethanolamine from serine directly (Mudd and Datko, 1989; Rhodes and Hanson, 1993). Indeed, the enzyme serine decarboxylase (SDC), which converts serine to ethanolamine in one reaction step, was found in *Arabidopsis thaliana* and rapeseed (Rontein et al., 2001). This enzyme has an important function in plant growth and development, because a leaky mutant of *A. thaliana* SDC1 displays multiple growth defects (Kwon et al., 2012) and a knockout mutant is embryonic-lethal (Yunus et al., 2016). In addition, overexpression of *SDC1* in tobacco increases ethanolamine content (Rontein et al., 2003), and in *A. thaliana* increased the contents of PC and PE besides ethanolamine in rosette leaves and mature siliques (Yunus et al., 2016). Thus, SDC1 catalyzes an important initial step of polar head group biosynthesis for the production of primary membrane phospholipids. However, the substrate, serine, is an amino acid that has multiple metabolic fates other than being a precursor for the biosynthesis of PC and PE (Ros et al., 2014). The endoplasmic reticulum (ER) is the primary organelle for the production of most phospholipid classes but not amino acids (Vance, 2015). It remains elusive how serine usage by SDC1 for phospholipid biosynthesis affects amino acid metabolism.

Here, using a transgenic *A. thaliana* plants expressing SDC1-Venus fusion protein in *sdc1-2* homozygous mutant background, we found that SDC1 was localized in mitochondria. Moreover, we showed that overexpression of *SDC1*, which increases PC and PE, decreased the levels of amino acid compounds derived from mitochondrial tricarboxylic acid (TCA) cycle. These results suggest that SDC1 plays an important role in both phospholipid and amino acid metabolism, and that an initial reaction step of phospholipid biosynthesis involves mitochondria.

## Materials and Methods

### Plant Materials and Growth Conditions

*A. thaliana* (ecotype; Columbia-0) was used. Plants were grown under long-day (16 h light/8 h dark) light condition at 22°C. For plate culture, Murashige and Skoog (MS) medium were used at half-strength concentration (Murashige and Skoog, 1962). The transgenic plant *ProSDC1:SDC1-Ven sdc1-2/-* line No. 13, *Pro35S:SDC1 sdc1-2/-* lines No. 1 and 7, and heterozygous *sdc1-2/+* mutant were as described previously (Yunus et al., 2016).

### Microscopy Analysis

Nomarski (DIC) images of developing embryos were obtained as described previously (Lin et al., 2015). Fluorescence of SDC1-Ven in plant tissues was observed using a confocal laser microscope (LSM 510 Meta, Carl Zeiss, Jena, Germany) equipped with LCI Plan-Neofluar 63×/1.3-numerical aperture (NA) immersion, Plan-Apochromat 20×/0.8-NA, and Plan-Apochromat 10×/0.45-NA objectives. For plasma membrane staining, samples were immersed in 5 μg/mL of FM 4-64 (Molecular Probes, Invitrogen) for 5 min. After rinsing with phosphate-buffered saline (PBS), the stained seedlings were observed by using a confocal microscope. Images were captured by use of LSM 510 v3.2 confocal laser microscope (Carl Zeiss, Jena, Germany) with filters for Venus (514 nm laser, band-pass 520–555 nm), for lignin autofluorescence (405 nm laser, band-pass 420–480 nm), and for FM4-64 (514 nm laser, long-pass 650 nm). The staining method with MitoTracker was as described previously (Ishizaki et al., 2005) using 200 nM MitoTracker Orange dye (CM-H_2_TMRos, Thermo Fisher Scientific, Waltham, MA, United States) in MS medium at half-strength concentration for 15 min at room temperature. For MitoTracker staining in leaf, excised leaves were incubated with 1 μM of dye in MS medium at half-strength concentration for 1 h at room temperature. The signal was visualized by using the filter with band-pass of 565–615 nm. All images were merged with DIC images. Transverse sections of hypocotyl were obtained with a microslicer (DTK-1000, Dosaka, Japan) in 100-μm thickness.

### Amino Acid Analysis

Amino acid contents were analyzed according to the previously described method for ethanolamine assay (Yunus et al., 2016).

## Results

### Defective Embryo Development Was Complemented in *ProSDC1:SDC1-Ven sdc1-2/-* Plants

**FIGURE 1:**
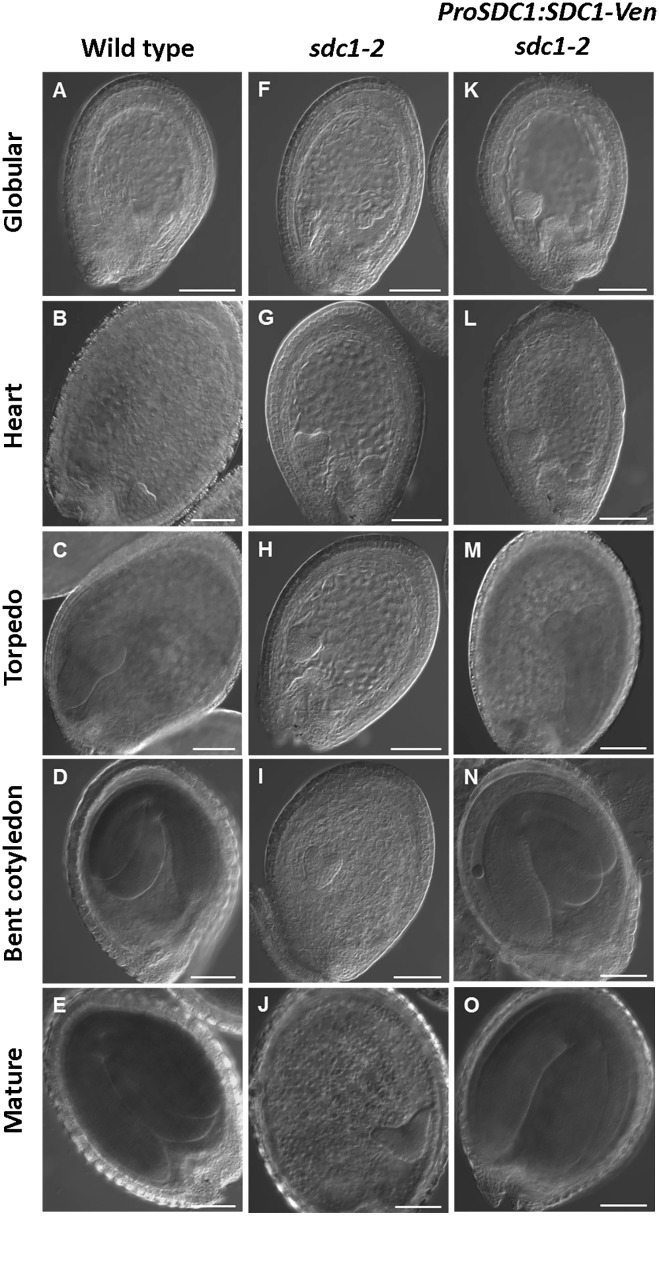
Microscopy analysis of embryo development in SDC1-Ven seeds. Normal wild type **(A–E)**, aborted embryos in *sdc1-2/+* **(F–J)**, and *ProSDC1:SDC1-Ven* *sdc1-2/–* embryos **(K–O)** at the stage of globular **(A,F,K)**, heart **(B,G,L)**, torpedo **(C,H,M)**, bent cotyledon **(D,I,N)**, and mature embryo **(E,J,O)**. Bars = 100 μm.

We previously showed that knocking out of *SDC1* causes an embryonic lethal phenotype in *sdc1-2/+* plants which produces 25% of albino seeds that contains underdeveloped embryos in siliques (Yunus et al., 2016). Although underdeveloped seeds were not found in the mutant carrying *ProSDC1:SDC1-Ven* transgene (*ProSDC1:SDC1-Ven sdc1-2/-* plant) (Yunus et al., 2016), it was unclear whether this transgenic plant demonstrates normal embryos at different developmental stages and thus functionally complements the embryo-lethal phenotype. We therefore observed morphology of embryos in a *ProSDC1:SDC1-Ven sdc1-2/-* plant at different developmental stages (**Figure 1**). In the *sdc1-2* seed, embryo development was arrested after the heart stage. In the *ProSDC1:SDC1-Ven sdc1-2/-* seed, however, this arrest was not observed and the seed development was indistinguishable from that of wild type throughout the development. This result indicates that *ProSDC1:SDC1-Ven* transgene is fully functional in rescuing the embryo-lethal phenotype of the *sdc1-2/-*. We therefore decided to use this line for subsequent localization study.

### Mitochondrial Localization of SDC1-Ven in Leaves and Roots

**FIGURE 2:**
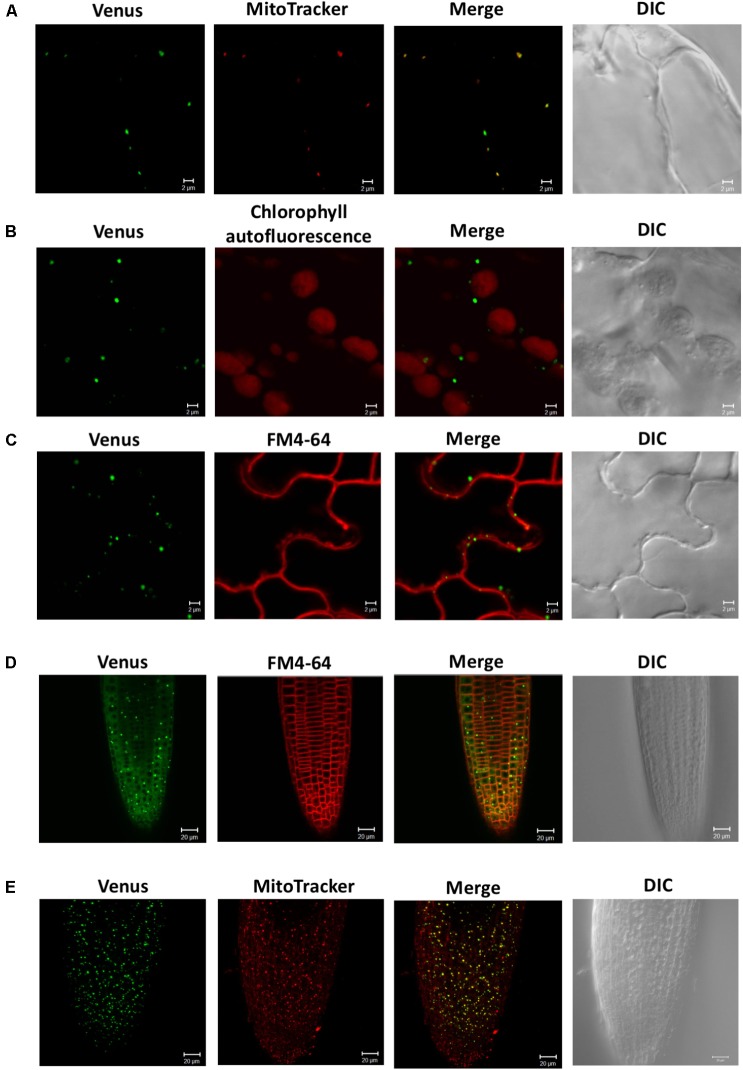
Subcellular localization of SDC1-Ven. **(A–C)** Confocal microscopy observation of SDC1-Ven in leaves. Expression of SDC1-Ven and staining pattern with MitoTracker **(A)**, chlorophyll autofluorescence **(B)**, and FM4-64 for plasma membrane marker **(C)** were merged. Bars = 2 μm. **(D,E)** Expression of SDC1-Ven in root tip and its co-localization with the staining pattern of FM4-64 for plasma membrane marker **(D)** or MitoTracker for mitochondrial marker **(E)**. Bars = 20 μm.

To investigate the subcellular localization of SDC1, we observed the fluorescence signal of SDC1-Ven in leaves and roots of *ProSDC1:SDC1-Ven sdc1-2/-* plants. In leaf epidermal cells, the fluorescence signal showed a clear overlap with MitoTracker for mitochondrial marker (**Figure 2A**) but not with chlorophyll autofluorescence (**Figure 2B**) or FM4-64 for the plasma membrane marker (**Figure 2C**). Next, in root tips, we observed particular signals in addition to a weak but ubiquitous fluorescence signal surrounding the nuclei (**Figure 2D**), which did not overlap with the plasma membrane marker dye (FM4-64). These fluorescent particles clearly overlapped with the staining pattern of MitoTracker (**Figure 2E**), suggesting mitochondrial localization of SDC1. These results suggest that SDC1 is localized in mitochondria of both leaves and roots.

### Expression of SDC1-Ven in Roots and Hypocotyls

**FIGURE 3:**
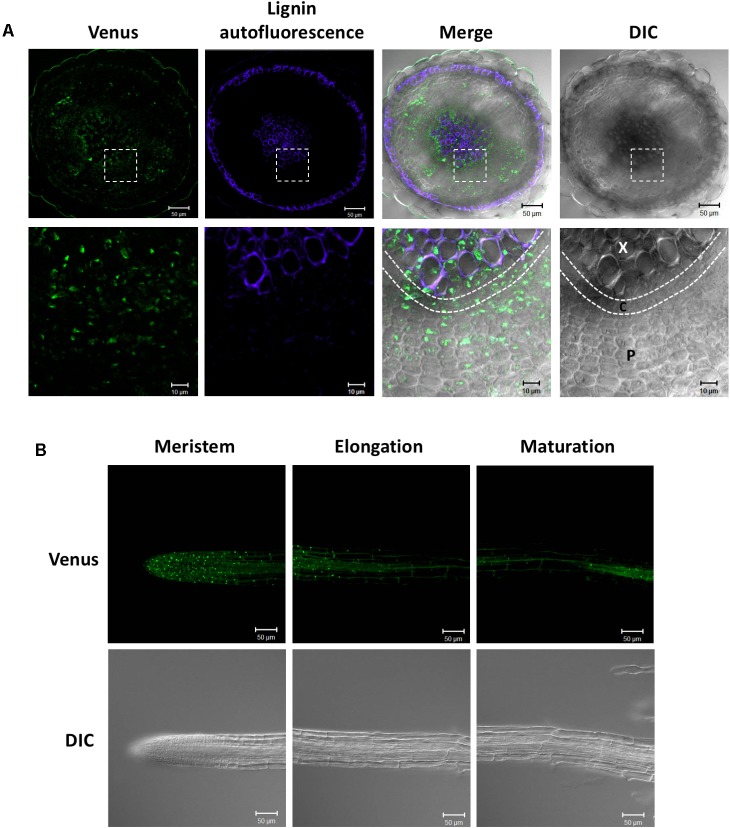
Expression of SDC1-Ven in hypocotyls and roots. **(A)** Expression of SDC1-Ven in transverse section of a 28-day-old hypocotyl of *ProSDC1:SDC1-Ven* *sdc1-2/–* plants. Bars = 50 μm (Upper); Bars = 10 μm (Lower). X, xylem; C, cambium; P, phloem. **(B)** Expression of SDC1-Ven in meristem, elongation, and maturation zones of 10-day-old main root of *ProSDC1:SDC1-Ven* *sdc1-2/–* plants. Bars = 50 μm.

We previously studied the expression pattern of SDC1-Ven in developing embryos (Yunus et al., 2016). In addition to the embryos, SDC1 is preferentially expressed in the vascular tissues. Because a leaky mutant of *SDC1* shows multiple growth defects (Kwon et al., 2012), an essential function of SDC1 may not be limited to the embryo development but to the other part of plant body including the vascular tissues. In order to further dissect the vascular expression pattern in the vegetative tissues, we observed fluorescence signal of SDC1-Ven in hypocotyls and roots of *ProSDC1:SDC1-Ven sdc1-2/-* plants. A transverse section of hypocotyl showed the fluorescent signal surrounds lignified cells, where secondary growth has occurred (**Figure 3A**). In addition, a considerable strength of fluorescent signal was confined at around the position where phloem is localized. Thus, SDC1-Ven is expressed both in xylem and phloem.

Next, we observed the fluorescence signal in the roots. In the maturation zone, the fluorescence was observed only in the vasculature, which is in agreement with the previous observation using GUS reporter gene (Yunus et al., 2016; **Figure 3B**). However, in the meristem zone and younger part of elongation zone, many small particle-like structures were observed, suggesting mitochondrial localization of SDC1 in this cell type.

### Effect of Overexpressing *SDC1* on Serine Content

**FIGURE 4:**
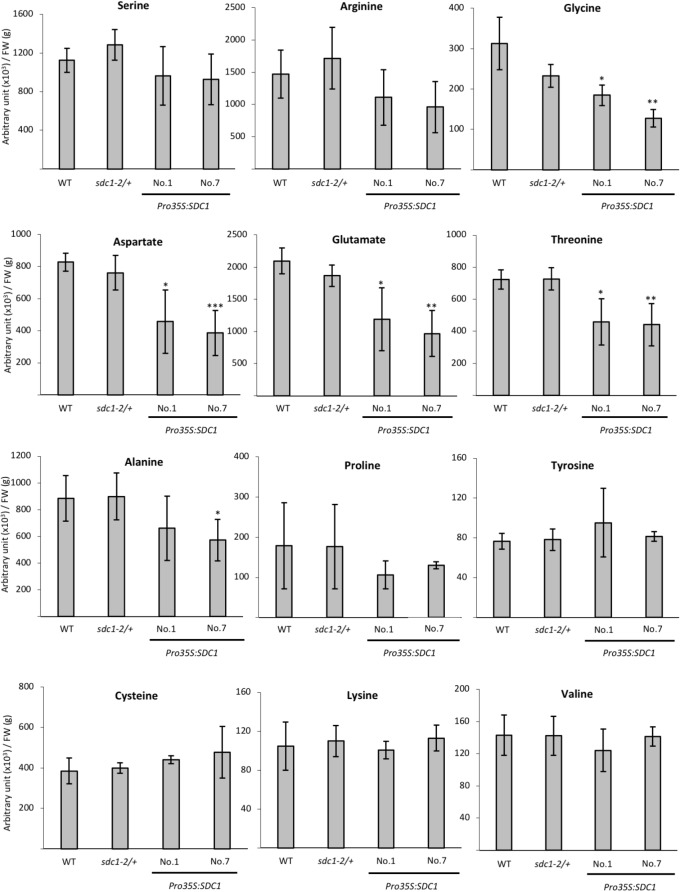
Contents of serine and other amino acids in rosette leaves of wild type (WT), *sdc1-2/+, Pro35S:SDC1* lines No. 1 and 7. Data are normalized to tissue fresh weight (FW) and shown as relative values of mean ± SD from four biological replicates. Statistical analysis was carried out by Student’s *t*-test (^∗^*P* < 0.05; ^∗∗^*P* < 0.01; ^∗∗∗^*P* < 0.001).

We previously showed that constitutive overexpression of *SDC1* by cauliflower mosaic virus 35S promoter in *A. thaliana* increases contents of ethanolamine, PE, and PC in rosette leaves (Yunus et al., 2016). However, it is unknown whether the overexpression of *SDC1* conversely decreases content of the substrate serine. We quantified the serine content in the rosette leaves of two independent lines of *SDC1* overexpression plants to assess the impact on the serine content. As shown in **Figure 4**, no significant change was observed as compared to the wild type or heterozygous mutant of *sdc1-2* (*sdc1-2/+*). This indicates that the increase in PC and PE contents by overexpressing *SDC1* do not alter the serine content.

### Amino Acid Profiles of *Pro35S:SDC1* Plants in Rosette Leaves

Because serine content was not changed by overexpressing *SDC1* in rosette leaves, we wondered whether contents of any other amino acid compounds could be affected. Thus, we analyzed contents of selected amino acid species in the rosette leaves of two independent lines of *SDC1*-overexpressing plants as well as wild type and *sdc1-2* heterozygous mutant. As shown in **Figure 4**, we found that contents of TCA cycle-derived amino acids (aspartate, glutamate, and threonine) showed significant and consistent decrease between two lines of *SDC1* overexpression plants. We found that glycine content was decreased whereas cysteine content was unaffected in the overexpressors. In addition, contents of the other TCA cycle-derived amino acids (lysine, glutamine, arginine, and proline) were unaffected. Thus, overexpression of *SDC1* increases contents of ethanolamine, PC, and PE but maintains serine level at the expense of other amino acid compounds. This suggests a possible cross talk between phospholipid biosynthesis and amino acid metabolism.

## Discussion

**FIGURE 5:**
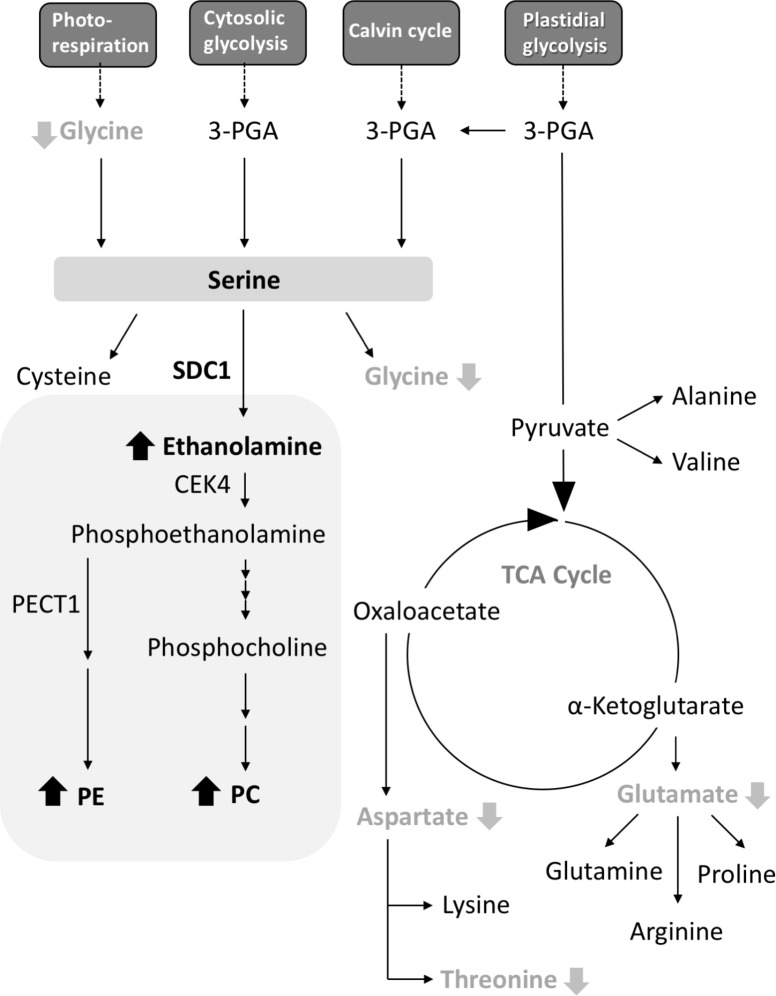
A schematic summary of metabolite changes occurred in rosette leaves of *Pro35S:SDC1* plants. Compounds in black bold letters with upward arrows and gray bold letters with downward arrows indicate increases and decreases in the *Pro35S:SDC1* plants compared to the wild type, respectively. Gray background indicates phospholipid biosynthesis pathway. 3-PGA, 3-phosphoglycerate; CEK4, choline/ethanolamine kinase 4; PC, phosphatidylcholine; PE, phosphatidylethanolamine; PECT1, CTP:phosphorylethanolamine cytidylyltransferase 1; SDC1, serine decarboxylase 1; TCA, tricarboxylic acid.

*A. thaliana* SDC1 catalyzes the first reaction step of the biosynthesis of polar head groups for PC and PE, and is an essential enzyme (Rontein et al., 2001; Yunus et al., 2016). The substrate serine is also a precursor for the biosynthesis of amino acids, so SDC1 activity may also affect amino acid metabolism. However, the effect of SDC1 activity on amino acid metabolism remained elusive. Here, our results indicate that SDC1 localizes in mitochondria and affects contents of several amino acids (**Figure 5**). Decrease in glycine content could be explained by an effect of increases in ethanolamine and phospholipids (PC and PE) content due to the overexpression of *SDC1* on the photorespiration pathway-derived serine biosynthesis, since this pathway is known to be the most important for serine biosynthesis in photosynthetic tissues (Tolbert, 1980; Douce et al., 2001). It is unknown why contents of aspartate, threonine, and glutamate, which are derived from mitochondrial TCA cycle, were reduced but the amount of the other TCA cycle-derived amino acids (lysine, glutamine, arginine, and proline), as well as that of cysteine derived from serine, were unaffected in *SDC1* overexpression lines. An underlying mechanism of changes in these amino acid contents may involve a complex metabolic interplay between different organelles.

Serine decarboxylase 1 was found as a soluble protein (Rontein et al., 2001). This is in agreement with the fact that SDC1 protein has no predictable transmembrane region. The result of prediction of subcellular localization by WoLF PSORT program^1^ gives a significant score of cytoplasmic localization (cyto 6). However, the effect of *SDC1* overexpression on the levels of amino acid compounds derived from mitochondrial TCA cycle suggests that SDC1 may be localized in proximity to mitochondria. Here, our results on the mitochondrial localization of SDC1 provide an intriguing insight into the subcellular localization of phospholipid biosynthesis. It is known that PC and PE are produced in the ER. However, because SDC1 localizes in mitochondria, it is possible that the initial reaction steps of PC and PE biosynthesis may occur outside of the ER. Interestingly, CTP:phosphorylethanolamine cytidylyltransferase 1 (PECT1), an essential enzyme for PE biosynthesis, also localizes in mitochondria (Mizoi et al., 2006). Although choline/ethanolamine kinase 4 (CEK4), which catalyzes the reaction step in between the reactions catalzyed by SDC1 and PECT1, is localized at the plasma membrane and also could be in the ER (Lin et al., 2015), it is possible that mitochondria are involved in the initial steps of polar head group biosynthesis for PC and PE. In future effort, it is important to elucidate the subcellular localization of entire pathway of PC and PE biosynthesis by studying the subcellular localization of the rest of enzymes (phospho-base *N*-methyltransferase, CTP:phosphorylcholine cytidylyltransferase; amino alcohol aminophosphotransferase) involved in this pathway (Bolognese and McGraw, 2000; Inatsugi et al., 2002; Liu et al., 2015). In conclusion, our results indicate that Arabidopsis SDC1 localizes in mitochondria and affects the content of a specific subset of amino acid species in addition to primary phospholipid classes.

## Author Contributions

YN conceived the research, supervised the experiment, and wrote up the research. Y-cL, FG, and IY performed the experiment and analyzed the data. All authors read the manuscript and approved the contents.

## Conflict of Interest Statement

The authors declare that the research was conducted in the absence of any commercial or financial relationships that could be construed as a potential conflict of interest.

## Footnotes

1 https://wolfpsort.hgc.jp/

## Abbreviations

CTP: cytidine triphosphate
ER: endoplasmic reticulum
PC: phosphatidylcholine
PE: phosphatidylethanolamine
SDC: serine decarboxylase
TCA: tricarboxylic acid.
